# Deep Learning for Ear-EEG-Based Brain–Computer Interface: A Systematic Comparison and Design Insights

**DOI:** 10.3390/bios16080437

**Published:** 2026-08-12

**Authors:** Ji-Seung Kim, Soo-In Choi, Han-Jeong Hwang, Chang-Hee Han

**Affiliations:** 1 Department of Computer and Information Science, Korea University, Sejong 30019, Republic of Korea; toiro@korea.ac.kr; 2 Medical Metrology Group, Division of Biomedical Metrology, Korea Research Institute of Standards and Science (KRISS), Daejeon 34113, Republic of Korea; sichoi@kriss.re.kr; 3 Department of Electronics and Information Engineering, Korea University, Sejong 30019, Republic of Korea; 4 Digital Healthcare Center, Sejong Institute for Business and Technology, Korea University, Sejong 30019, Republic of Korea; 5 Interdisciplinary Graduate Program for Artificial Intelligence Smart Convergence Technology, Korea University, Sejong 30019, Republic of Korea; 6 Department of Computer Science and Software Engineering, Korea University, Sejong 30019, Republic of Korea

**Keywords:** Ear-EEG, brain–computer interface, mental imagery, deep learning architecture, lightweight convolutional neural network

## Abstract

Electroencephalography (EEG) measured inside or around ears, called ear-EEG, provides a practical measurement modality for daily brain–computer interface (BCI) applications. However, reliable decoding of mental imagery remains challenging due to the limited number of channels, low signal-to-noise ratio (SNR), and substantial inter- and intra-subject variability inherent to ear-EEG. Addressing these constraints requires advanced decoding strategies specifically optimized for this signal domain. In this study, we retrospectively analyze the ear-EEG dataset of a previous study in which a real-time endogenous BCI was evaluated using conventional machine learning. Specifically, we present an offline benchmark of 23 deep neural network architectures originally developed for scalp-EEG, which were adapted to ear-EEG and evaluated under an identical validation framework. To the best of our knowledge, this is the first systematic comparison of this breadth for ear-EEG-based mental-task classification. Beyond conventional performance comparison, we identify the optimal architecture by jointly considering statistical significance and a performance–cost trade-off, incorporating classification accuracy, parameter count, and measured computational cost. Our results demonstrate that FBLightConvNet achieves the highest classification accuracy among all evaluated models and outperforms common spatial pattern-linear discriminant analysis (CSP-LDA), a widely adopted and robust conventional baseline, on all three recording days, with the difference reaching statistical significance on Days 2 and 3. Notably, many state-of-the-art scalp-EEG models fail to generalize effectively to ear-EEG, highlighting the importance of architecture selection in this domain. These findings identify the best-performing architecture in this setting and indicate which architectural characteristics support effective ear-EEG decoding. Ultimately, this study offers practical design insights and a reproducible benchmarking framework for developing lightweight and high-performance deep learning models, which we hope will support future efforts toward real-world ear-EEG-based BCI systems.

## 1. Introduction

Brain–computer interfaces (BCIs) translate neural activity into actionable commands [1]. By bypassing peripheral neuromuscular pathways, these BCI systems provide an intuitive communication channel for users to convey their intentions through distinct brainwave modulations without requiring overt physical movement or speech [2]. Various protocols exist to operate these BCI systems. Mental imagery, particularly the kinesthetic imagination of left- or right-hand movements, has emerged as a primary control mechanism for driving robotic prosthetics [3], motorized wheelchairs, and virtual spellers [4]. This endogenous strategy offers distinct physiological advantages. Whereas exogenous protocols such as P300 [5] and steady-state visual evoked potentials (SSVEP) [6] inherently rely on external stimuli and demand intact oculomotor control for continuous visual fixation, mental imagery operates entirely independent of physical movement [7]. Users generate commands solely through internal cognitive processes. Because this approach circumvents the need for even the slightest muscular exertion, including eye movements, it provides a viable communication channel for patients suffering from locked-in syndrome [8]. However, accurately decoding these cognitive states remains a major challenge, as classification algorithms must maintain high precision while being robust to signal noise, environmental interference, and significant inter-subject variability [9].

Early electroencephalography (EEG)-based BCIs designed for mental task decoding predominantly relied on traditional machine learning (ML) frameworks that utilize handcrafted features to translate raw neural signals into discrete commands. Among these ML-based techniques, common spatial patterns (CSPs) paired with linear discriminant analysis (LDA) emerged as a representative algorithm, demonstrating reliable classification performance [10,11]. Operating by maximizing the variance between distinct task conditions, CSP isolates discriminative spatial features, which the LDA classifier subsequently separates using a straightforward linear boundary [12]. Advanced variations, such as filter bank-based CSP, further refined this extraction process by incorporating frequency-specific spatial information [12]. These traditional ML methods have unfortunately shown several significant limitations. Especially, they demand exhaustive manual feature engineering and rely heavily on prior domain knowledge [13]. Because human brainwaves are inherently non-stationary, the generalization capability of such manually tuned algorithms frequently degrades when confronted with variable recording environments or inter-subject differences [14].

Recently, deep neural networks (DNNs) have been increasingly adopted in EEG signal processing to overcome the limitations of traditional ML approaches. These DNN architectures improve classification performance by jointly learning spatio-temporal and spectral information, effectively eliminating the need for manual feature engineering [15]. Among the various DNN models, convolutional neural network (CNN)-based models can simultaneously capture inter-channel spatial correlations and temporal dynamics, yielding significant performance improvements across various BCI tasks, including mental tasks [16]. Performance variations remain substantial, however, depending heavily on the specific model structure and data conditions [17]. Despite this critical dependency, comparative studies investigating the optimal architecture for specific signal types with a restricted channel count, such as ear-EEG, remain still limited.

Ear-EEG presents a practical alternative for implementing BCIs in everyday environments [18]. Placing electrodes around the ear allows for long-term wearability and high user convenience [19]. Nevertheless, ear-EEG suffers from limited electrode placement options compared to conventional scalp EEG. The structural characteristics of electrodes located in close proximity around the ear render both reference and recording sites susceptible to shared noise and reference signal effects [18]. Furthermore, spatial information is relatively weak due to the limited number of channels, making modeling strategies designed to secure reliable task classification performance critical. While existing studies indicate that certain CNN models outperform CSP-LDA-based methods for ear-EEG cognitive task classification [20], the scope of deep learning architectures compared in these studies is often limited.

The primary objective of this study is to investigate whether various deep learning models originally developed for scalp-EEG can demonstrate superior performance on ear-EEG data, and to derive critical insights for designing ear-EEG-specific models through a systematic comparative analysis. To achieve this, we collectively compared 23 deep neural network models originally designed for scalp-EEG classification using an identical ear-EEG dataset and evaluation protocol. Moving beyond simple average accuracy comparisons, we identified the optimal deep learning model by integrating statistical significance testing with a comprehensive analysis of a performance-complexity metric based on the number of parameters for each DL model. Finally, we analyze the architectural trends of deep learning models best suited for ear-EEG mental imagery classification from the perspective of model structure and computational efficiency. The dataset, the acquisition system, the mental-task paradigm, the three recording days, and the subject-specific task selection were all established in our previous study [21], in which a real-time endogenous ear-EEG BCI was evaluated using CSP-LDA. No new recordings and no new online experiments are reported here. The present study is instead a retrospective offline benchmark of existing deep learning architectures conducted on those data, from which we derive the accuracy ranking of the 23 DNN architectures, the relationship of this ranking to architectural family and computational cost, and a reproducible evaluation framework for ear-EEG decoding.

## 2. Materials and Methods

### 2.1. Data Acquisition and Preprocessing

The ear-EEG data used in this study are the same as presented in our previous study [21], where we evaluated the feasibility of a real-time endogenous BCI system using ear-EEG. Fourteen participants (8 males and 6 females; age: 25.6 ± 1.7 years) with normal or corrected-to-normal vision and hearing were enrolled, and they participated in the experiment three times on different days. None of the participants reported a history of neurological disorders. Written informed consent was obtained prior to the experiment. The Institutional Review Board of Kumoh National Institute of Technology approved the study (IRB No. 6250), which was conducted in compliance with applicable guidelines. Participants sat comfortably approximately 1 m in front of a computer monitor, instructed to stay relaxed with eyes open while minimizing eye and body movements to reduce muscle-related artifact contamination.

**Figure 1:**
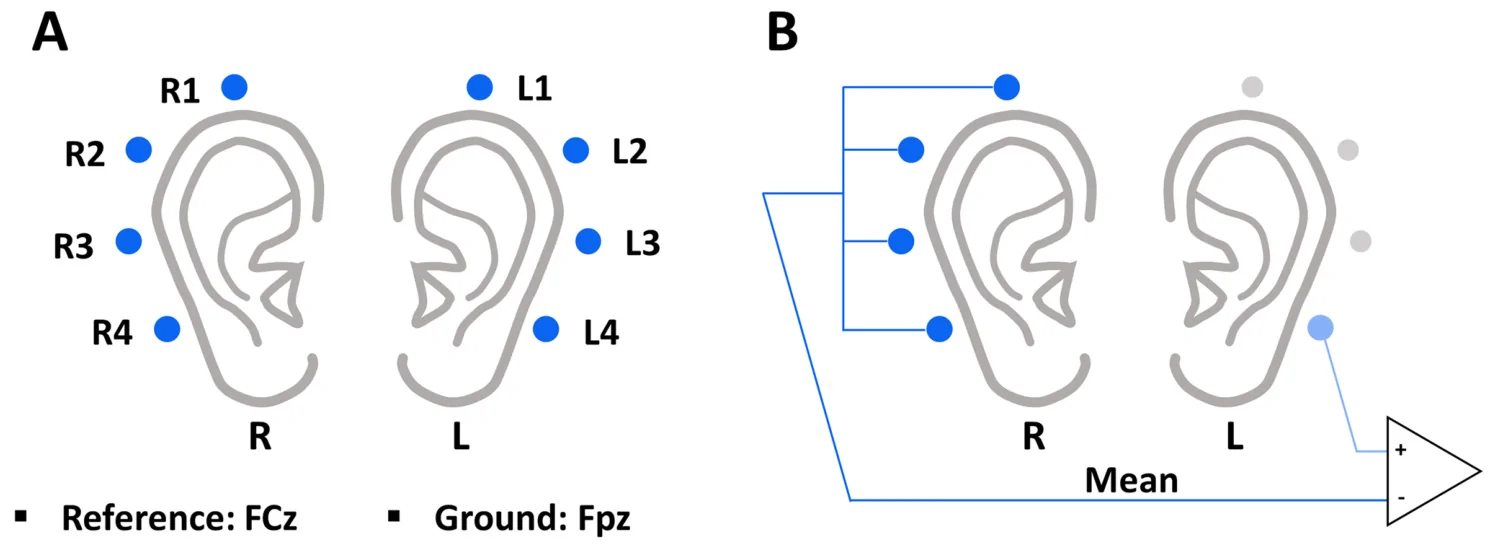
Ear-EEG electrode configuration and spatial re-referencing method. (**A**) Spatial layout of the eight retroauricular ear-EEG electrodes (L1–L4 behind the left ear; R1–R4 behind the right ear) and the reference and ground electrodes based on the 10–20 system. (**B**) Schematic representation of the contralateral mean re-referencing approach, where the average signal of the opposite ear’s electrodes is subtracted from each respective channel. Blue circles indicate the ear-EEG electrodes. In (**B**), the dark blue circles denote the contralateral electrodes whose signals are averaged, the light blue circle denotes the channel being re-referenced (L4 is shown as an example), and the gray circles denote the remaining electrodes of the same ear. Redrawn and adapted from [21], which is published under the Creative Commons Attribution (CC BY) license.

Ear-EEG signals were recorded using an active-electrode EEG system (actiCHamp; Brain Products GmbH, Gilching, Germany). As illustrated in Figure 1A, eight ear-EEG electrodes were configured in the retroauricular regions (behind both ears). Specifically, channels L1, L2, L3, and L4 were positioned behind the left ear, while R1, R2, R3, and R4 were positioned behind the right ear. In accordance with the international 10–20 system, Fpz and FCz served as the ground and reference electrodes, respectively [22]. Electrode impedances were maintained below 10 kΩ to ensure high signal quality and minimize environmental noise [23], and raw data were recorded at 1000 Hz of sampling frequency.

To minimize noise in the raw ear-EEG signals, an offline preprocessing pipeline was implemented using MATLAB R2025a (MathWorks Inc., Natick, MA, USA). First, a 1–50 Hz bandpass filter using a zero-phase sixth-order Butterworth filter was applied to the data from each session. The filtered data were subsequently down-sampled to 200 Hz. To further isolate localized neural activity, the signals were spatially re-referenced using the mean of the contralateral ear-EEG channels (Figure 1B) [24]. Finally, the continuous signals were segmented into 10 s epochs (0 to 10 s) relative to the mental task onset for subsequent classification.

### 2.2. Experimental Protocols

In this study, we used a dataset from our previous study [21]. Data were collected across three experimental days. On Day 1, an optimal pair of mental tasks was selected from three candidate tasks; specifically, this was the combination that yielded the highest classification accuracy for each subject when applying the CSP-LDA algorithm. Subsequently, online experiments were conducted on Days 2 and 3 to evaluate individually optimized task-pair selection for an endogenous ear-EEG BCI. Although the previous study included online BCI implementation on Days 2 and 3, in the present study, all collected data were exclusively used for offline analysis to investigate the performance of various deep learning models. The experimental protocol summarized in this section is that of Ref. [21].

On Day 1, participants performed three mental imagery tasks: mental arithmetic (MA), mental singing (MS), and word association (WA). Task order was randomized to prevent participants from anticipating the upcoming tasks. Each session comprised 10 trials per task. Five sessions were completed, yielding 50 trials for each task. Classification performance was computed for all three binary task pairs (MA vs. MS, MA vs. WA, and MS vs. WA) using the Day 1 data. The task pair exhibiting the highest performance for each participant was selected and used exclusively during the subsequent two days of experiments (Days 2 and 3).

**Figure 2:**
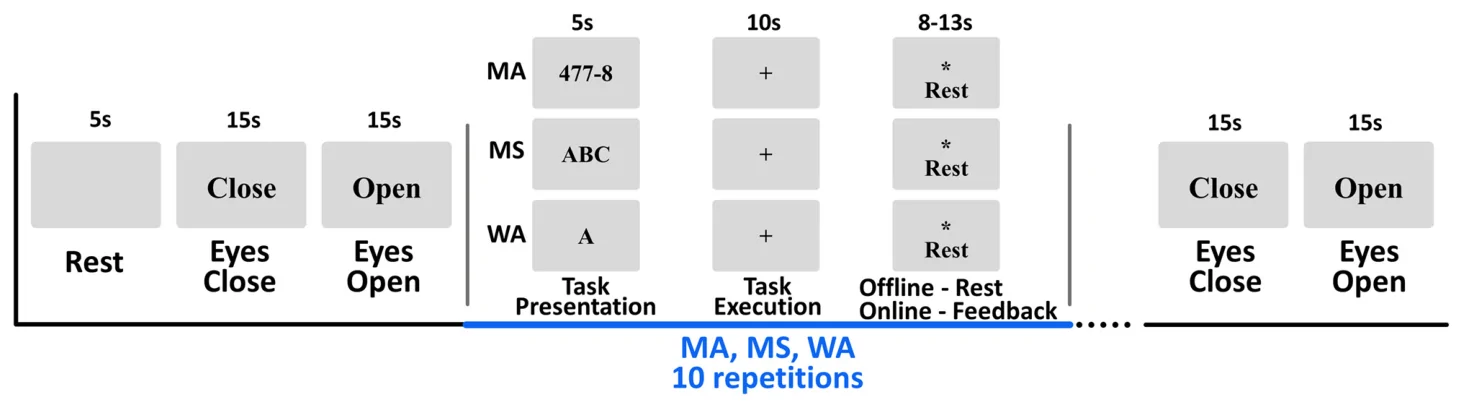
Schematic diagram of the experimental paradigm. The asterisk denotes the symbol presented on the screen during the rest period and does not indicate statistical significance. Redrawn and adapted from [21], which is published under the Creative Commons Attribution (CC BY) license.

Figure 2 shows the detailed experimental paradigm of this study. Each session began with a 5 s blank screen, followed by the visual cues “Close” and “Open” presented sequentially for 15 s each to instruct eye closures and openings. Each experimental trial consisted of a 5 s task-cue period, a 10 s task-execution period, and a randomly jittered rest period of 8–13 s. Task cues were delivered as pictograms displayed at the center of the screen. These included a subtraction problem (e.g., “477 − 8”) for MA, the string “ABC” for MS, and a single letter (e.g., “A”) for WA. Participants performed the instructed mental task while fixating on the screen to minimize eye movements during the 10 s execution window. For MA, participants repeatedly subtracted a small integer (6–9) from an initial three-digit number and continued subtracting the same integer from each subsequent result. For MS, participants silently imagined an English alphabet song at a rate of approximately 1 Hz. For WA, participants generated as many words as possible beginning with the presented letter. An asterisk and the word “Rest” were shown during the 8–13 s rest interval to reduce habituation. Participants were instructed to relax and avoid deliberate thinking during this period, reflecting a typical no-control state in BCI operation. Each daily session lasted approximately 2 h, including setup and scheduled breaks.

### 2.3. Evaluation and Selection of the Optimal Deep Learning Model

For the offline deep learning analysis, the preprocessed ear-EEG data were standardized using an exponential running standardization method [25]. We evaluated 23 DL models provided by the Braindecode open-source toolbox for scalp-EEG decoding based on PyTorch [26]. These included EEGSimpleConv [27], FBLightConvNet [28], EEGInceptionMI [29], IFNet [30], MSVTNet [31], FBCNet [32], FBMSNet [33], AttentionBaseNet [34], EEGNeX [35], SCCNet [36], ShallowFBCSPNet [26], EEGMiner [37], SincShallowNet [38], SPARCNet [39], ATCNet [40], EEGTCNet [41], Deep4Net [26], CTNet [42], EEGInceptionERP [43], EEGITNet [44], EEGConformer [45], TIDNet [46], and SyncNet [47]. The classification accuracy was computed using a subject-specific stratified 10-fold cross-validation. This scheme was applied independently for each subject, recording day, and mental task pair, so that trials were never mixed across subjects, days, or task pairs, thereby preventing any cross-subject or cross-day leakage. For a given subject, day, and task pair, the trials were divided in a class-stratified manner into 10 folds. Days 1 and 2 comprised five sessions and 100 trials per task pair, whereas Day 3 comprised nine sessions and 180 trials, because in Ref. [21] the two online test sessions of Day 3 were repeated three times with classifiers built from different training data. In each split, one fold (10%) was held out as the test set, and the remaining trials were used for training (≈81%) and validation (≈9%). The validation subset served only for early stopping, whereas the held-out test fold was used exclusively for performance evaluation. The subject-level accuracy was computed as the mean over the 10 folds, and the reported accuracies represent the across-subject average. Each model was trained for a maximum of 250 epochs using a batch size of 64, the AdamW optimizer with a learning rate of 0.005, and a cross-entropy loss. Early stopping was applied by monitoring the validation loss with a patience of 50 epochs and a guaranteed minimum of 40 epochs, after which the weights achieving the lowest validation loss were restored. To ensure a fair comparison across heterogeneous architectures, this shared training protocol was kept identical for all 23 models. No systematic hyperparameter search was performed. Exploratory adjustments of individual hyperparameters during preliminary work did not produce meaningful changes in accuracy, and every model was therefore evaluated with the default configuration provided by Braindecode, that is, the configuration recommended by its original authors, so that no architecture received more tuning than any other. The only exception is FBLightConvNet, for which the number of filter-bank bands was set to 18 rather than the default of 9. This value was obtained by exploratory adjustment on the present dataset rather than by a systematic search, and it is therefore specific to these data. As a control, FBLightConvNet was additionally evaluated at the default of nine bands using the identical pipeline, cross-validation folds, and seeds. Its overall accuracy was 75.02%, and it retained the highest accuracy among the 23 architectures, ahead of EEGSimpleConv at 73.60%. The architecture ranking reported in this study is therefore preserved under the default configuration and does not depend on this single non-default setting. All architectures received the same input tensor, namely the eight retroauricular channels of a 10 s epoch sampled at 200 Hz. No channel or temporal transformation was applied for individual models. The filter-bank architectures decompose this input internally into sub-bands spanning 4–40 Hz as the first operation of their forward pass, using two bands for IFNet, nine each for FBCNet and FBMSNet, and 18 for FBLightConvNet. Analyses were performed with Python 3.10.18 (Python Software Foundation, Wilmington, DE, USA), PyTorch 2.11.0 (PyTorch Foundation, San Francisco, CA, USA), CUDA 12.8 and cuDNN (NVIDIA Corporation, Santa Clara, CA, USA), Braindecode 1.2.0 [26], and MNE-Python 1.12.1 (https://mne.tools) on an NVIDIA GeForce RTX 5080 graphics processing unit (NVIDIA Corporation, Santa Clara, CA, USA) with 16 GB of memory, with cuDNN in deterministic mode.

To quantify run-to-run variability, the entire evaluation of the seven leading architectures was repeated with three random seeds. Each seed is a triple that sets the cross-validation partition, the train/validation split within each training fold, and the network weight initialization, so that the reported variability reflects more than initialization alone. The specific values are given in the configuration files of the released code. The accuracies reported elsewhere in the manuscript correspond to the first seed, and the mean and standard deviation across the three seeds are reported in Section 3.4.

To characterize computational cost beyond parameter count, we additionally measured, for each architecture, the number of multiply–accumulate operations required for a single trial, the peak memory used during inference, and the single-trial inference time. Multiply–accumulate operations were counted with the PyTorch operator-level FLOP counter, which registers convolutions, matrix multiplications, and attention operations; element-wise activations, normalization, and pooling are not included, following the usual convention. Inference time was measured on real preprocessed trials as the mean of 200 timed single-trial forward passes after 30 warm-up passes, with CUDA synchronization before and after each measurement, and is reported for the graphics processing unit described above. Peak memory was recorded during a single-trial forward pass. All measurements used the input dimensions and model configurations of the classification experiments, so that the reported cost corresponds to the reported accuracy.

Statistical analysis was performed separately for each of the five conditions, namely the three Day 1 task pairs and the subject-specific optimal pair on Days 2 and 3. Within a condition, the ten cross-validation accuracies of each participant were averaged into a single value, so that the statistical unit was the participant (*n* = 14) and correlated folds were not treated as independent observations. A Friedman test was applied across the 23 architectures, with Kendall’s W reported as the effect size, followed by one-sided Wilcoxon signed-rank tests (Pratt treatment of zero differences) for all 23 × 22 = 506 ordered model pairs. The Benjamini–Hochberg false discovery rate correction was applied across the 506 tests within each condition; no correction was applied across conditions, which were analyzed separately. For each comparison, we additionally report the matched-pairs rank-biserial correlation, the Hodges–Lehmann median difference, and a bias-corrected and accelerated bootstrap 95% confidence interval for the mean difference. For each architecture, we counted the number of comparisons in which it was significantly more accurate than another architecture, and we additionally ranked the architectures by mean accuracy and by mean rank across participants. Subsequently, we evaluated the computational cost of the top seven DL models that achieved an accuracy exceeding 70%, which is considered a meaningful threshold for binary classification [48]. Through this process, the best DL model demonstrating both the highest accuracy and low computational cost was selected.

### 2.4. Evaluation Using the Original Training and Test Session Split

To examine whether the architecture ranking depends on the evaluation protocol, we additionally evaluated all 23 models using the session structure of the original study [21], which preserves the temporal and operational separation between training and test data. Day 2 comprised three training sessions followed by two online test sessions. Day 3 comprised three training sessions followed by the two test sessions repeated three times with classifiers built from different training data. Four splits were defined accordingly. Split 1 trained on Day 2 sessions 1–3 and tested on Day 2 sessions 4–5. Split 2 trained on Day 2 sessions 1–3 and tested on Day 3 sessions 4–5. Split 3 trained on Day 3 sessions 1–3 and tested on Day 3 sessions 6–7. Split 4 trained on Day 2 and Day 3 training sessions and tested on Day 3 sessions 8–9. In each split, the model was trained only on the training sessions, the validation subset used for early stopping was drawn exclusively from those training sessions, and the test sessions were never seen during training or model selection. All other settings were identical to those described in Section 2.3. Agreement with the pooled cross-validation was quantified by the rank correlation across the 23 architectures, since the unit of interest in this analysis is the architecture rather than the participant.

### 2.5. Performance Validation Against a Conventional Machine Learning Baseline

To further validate the best DL model’s performance, this optimal DL model was compared against a conventional baseline method, CSP-LDA, evaluated on the same dataset. The baseline consists of multi-band CSP for feature extraction and shrinkage LDA (sLDA) for classification [12], a combination that has demonstrated stable performance in ear-EEG studies [12]. Specifically, sLDA was used for binary classification based on features extracted via multi-band CSP (δ-band: 1–3 Hz, θ-band: 4–7 Hz, α-band: 8–13 Hz, β-band: 14–29 Hz, and γ-band: 30–50 Hz). To ensure a fair and direct comparison, the CSP-LDA pipeline used the same subject-specific task pair that was selected on the Day 1 data with CSP-LDA, following Ref. [21], and this pair was applied consistently across Days 1, 2, and 3. Finally, we evaluated the statistical significance of the classification performance difference between the best DL model and the CSP-LDA baseline using the Wilcoxon signed-rank test.

### 2.6. Cross-Dataset Evaluation of the Architecture Ranking

To examine whether the relative performance of the evaluated architectures is reproducible beyond the present dataset, we additionally benchmarked all 23 deep learning models on a second ear-EEG dataset that was acquired in a previous study for eyes-open versus eyes-closed classification [20]. This dataset was recorded from 30 participants, none of whom took part in the present study, on two separate recording days, using six retroauricular electrodes with three behind each ear rather than the eight used here, with 60 trials per participant per day. Both datasets were acquired by our own research group, so this analysis does not constitute external validation in the conventional sense of data collected at a different institution. The two datasets nonetheless differ in participants, recording period, electrode configuration, and task paradigm. To ensure a controlled comparison, the data were processed with exactly the same pipeline used for the main dataset, namely a 1–50 Hz zero-phase Butterworth band-pass filter, contralateral mean re-referencing, exponential moving standardization, and trial-wise input construction. Each model was trained and evaluated using the identical subject-specific stratified 10-fold cross-validation scheme and training configuration described in Section 2.3. For each model, the subject-averaged accuracy on this second dataset was computed by averaging the accuracies over its two recording days. The agreement between the two datasets was quantified using Pearson’s correlation coefficient and Spearman’s rank correlation coefficient computed across the 23 models, and the corresponding regression line was estimated by ordinary least squares. The subject-averaged accuracy of each architecture on each of the two recording days of this dataset is reported in Section 3.7. The unit of observation for these correlations is the architecture rather than the participant, so that they quantify the agreement of the architecture ranking between the two datasets. Because all models were retrained from scratch on the second dataset, this analysis also does not demonstrate transfer to unseen participants or validate mental-task decoding.

## 3. Results

### 3.1. Subject-Specific Optimization of Mental Task Pairs

Based on the Day 1 classification accuracy, the optimal mental task pair was individually determined for all 14 subjects. Table 1 illustrates the specific distribution of these selected pairs. Exactly half of the subjects (*n* = 7) achieved their highest accuracy with the MA vs. MS pair, while the remaining subjects were optimized with either MA vs. WA (*n* = 4) or MS vs. WA (*n* = 3).

### 3.2. Comparative Classification Performance of the 23 Deep Learning Models

**Figure 3:**
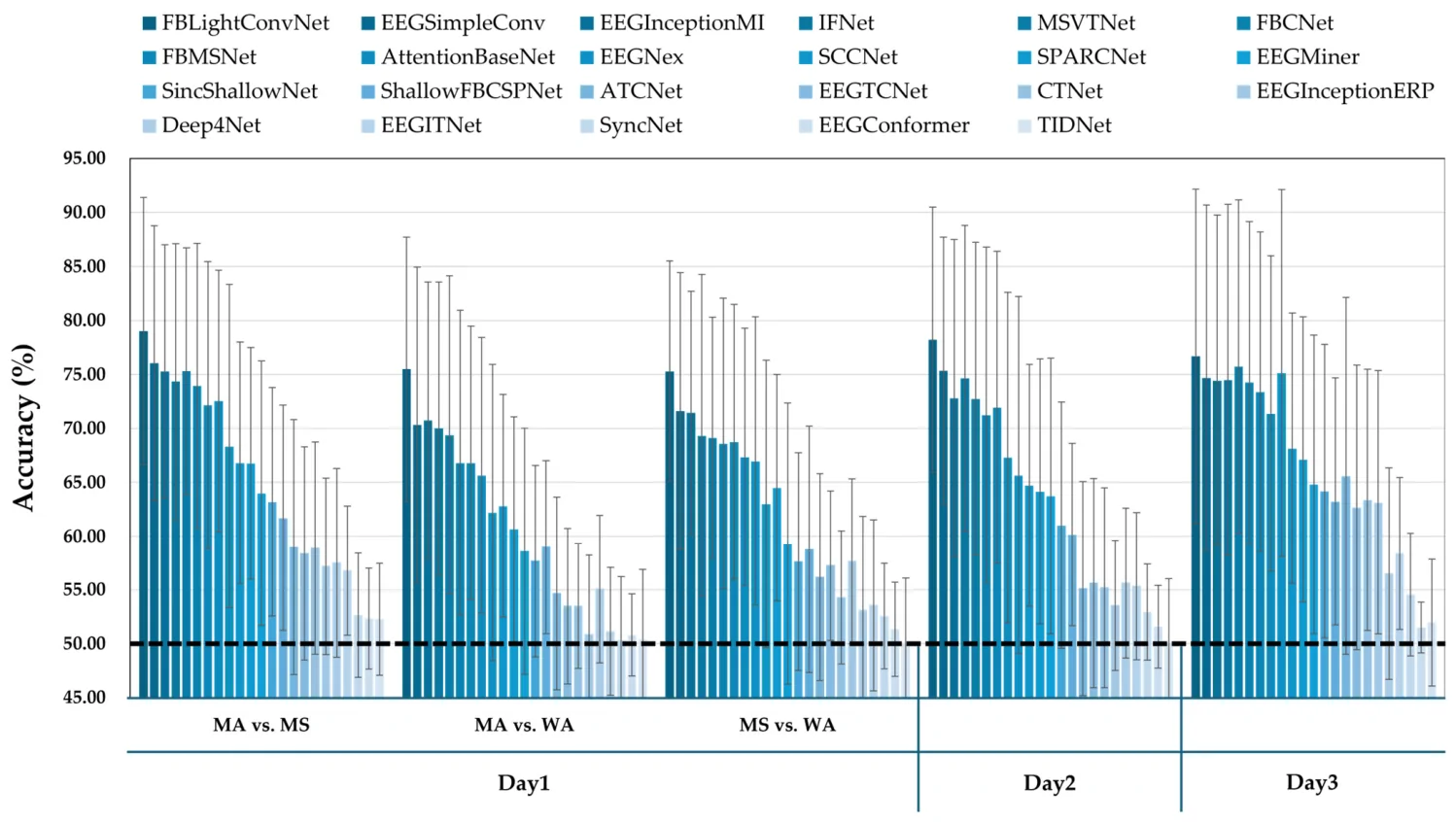
Comparison of mean classification accuracies across the 14 subjects for the 23 deep learning models. For Day 1, all three mental task pairs (MA vs. MS, MA vs. WA, and MS vs. WA) are shown, whereas for Days 2 and 3 the subject-specific optimal task pair selected on Day 1 is shown. Bars indicate the mean across subjects and error bars indicate the standard deviation across subjects. In the legend, the conditions are ordered from the first to the fourth row and, within each row, from left to right. The horizontal black dotted line indicates the theoretical chance level (50%).

To evaluate the efficacy of various model architectures for ear-EEG decoding, we comprehensively compared the classification accuracies of the 23 candidate DL models. Figure 3 presents the subject-averaged accuracies across all conditions, including the three mental task pairs on Day 1 and the selected optimal task pairs on Days 2 and 3. The results visually demonstrate a substantial variance in decoding performance depending on the model architecture. Among the 23 models, FBLightConvNet consistently achieved the highest subject-averaged accuracy across almost all conditions. Its peak performance was observed during the MA vs. MS task on Day 1, with an average accuracy approaching 80%. Overall, FBLightConvNet yielded the highest overall average accuracy of 76.94% across Days 1, 2, and 3 among all evaluated DL models. In contrast, 16 out of the 23 DL models exhibited a low average classification accuracy of less than 70%, a threshold commonly regarded as indicative of meaningful performance in binary classification [48]. Notably, some DL models, including Deep4Net, which is known for its excellent performance in motor imagery classification using conventional scalp-EEG, struggled to classify the ear-EEG-based mental tasks, yielding accuracies close to the chance level (~50%). Specifically, among the Day 1 task combinations, the MA vs. MS pair generally provided higher accuracies for the top-performing models compared to the MA vs. WA and MS vs. WA pairs. Finally, the performance of the leading models on Days 2 and 3 remained within a comparable range to their performance on Day 1 when an optimized DL model was used alongside subject-specific task pairs.

### 3.3. Pairwise Statistical Comparison and Identification of Top-Performing Models

To evaluate the performance differences among the 23 DL architectures, we conducted post hoc pairwise statistical comparisons of the classification accuracies. Specifically, for each DL model, we calculated the total number of times it demonstrated a statistically significant higher classification accuracy compared to the other 22 DL models. Table 2 provides a representative example of this pairwise statistical comparison, presenting the results derived from the MA vs. WA task condition on Day 1. In this table, the asterisks denote statistically significant differences between the DL models in the corresponding rows and columns based on the post hoc analysis, and the rightmost ‘Total’ column displays the total number of significant outperformance instances for each respective model. By systematically applying this evaluation across all combinations of recording days and mental task pairs, we compared the DL architectures for ear-EEG decoding.

**Figure 4:**
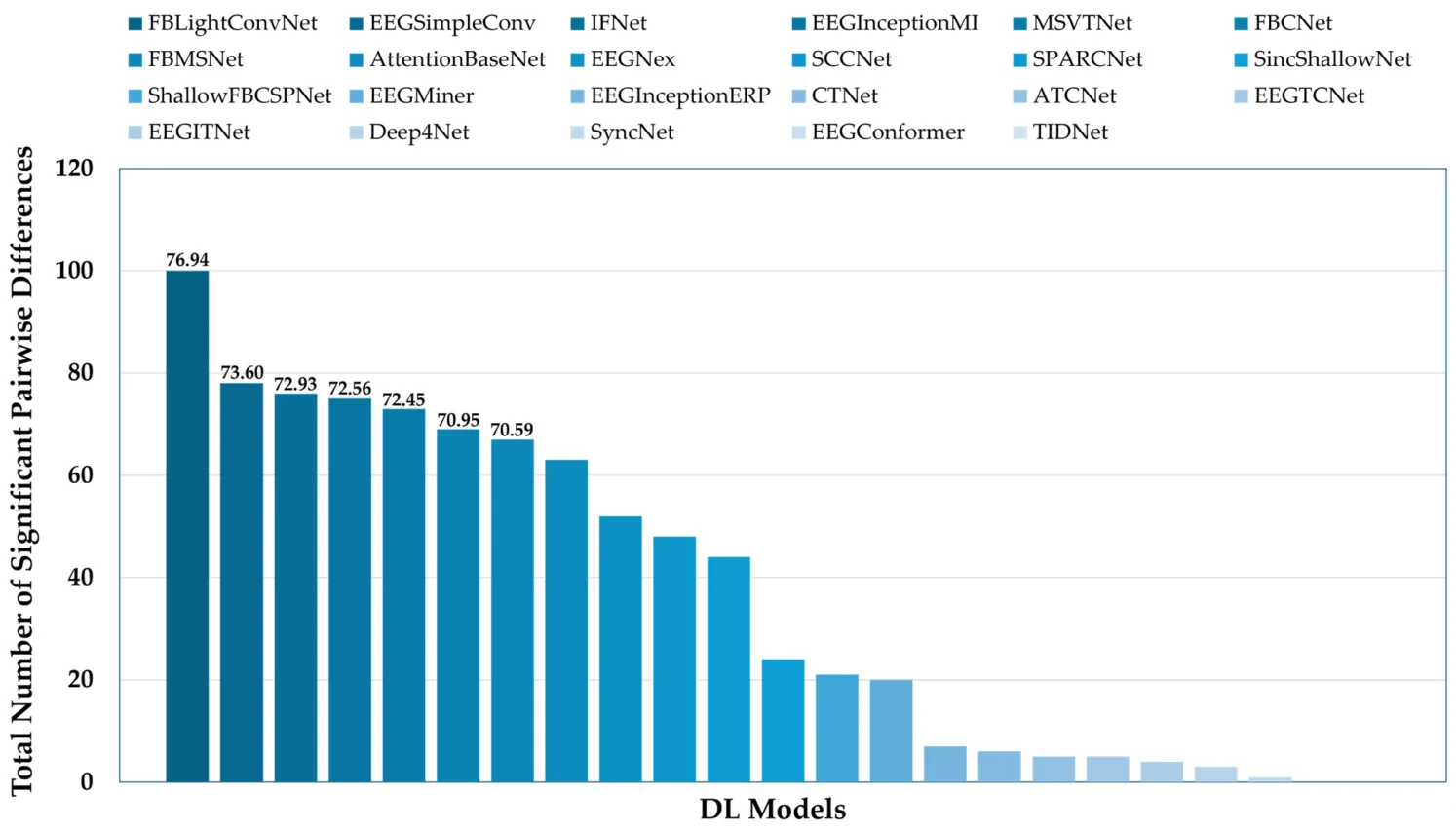
Cumulative Count of Significant Pairwise Differences for Each DL Model. The *y*-axis represents the total number of significant pairwise differences for each model, while the numbers displayed above the bars indicate their corresponding classification accuracies.

Figure 4 presents the total number of significant pairwise differences for each DL model, summed across all recording days on Days 1, 2, and 3. As shown in the results, only seven DL models achieved a classification accuracy above 70%, which is considered a meaningful threshold for binary classification [48]. Among these top DL models, FBLightConvNet demonstrated the highest performance, recording a cumulative count of 100 instances where it significantly outperformed the other DL models. On the other hand, the remaining 16 models failed to reach this 70% accuracy criterion. Interestingly, while all 23 models are well-known for their excellent performance in scalp-EEG, their classification accuracies varied considerably, ranging from approximately 50.98% to 76.94%, when applied to our ear-EEG dataset.

The omnibus test was significant in every condition with a large effect size, with Kendall’s W of 0.662, 0.476, 0.530, 0.582 and 0.559 for MA vs. MS, MA vs. WA, MS vs. WA, Day 2 and Day 3, respectively, and Friedman *p* below 1 × 10^−19^ in all five conditions. Across the 30 comparisons of FBLightConvNet against the other six leading architectures, the difference favored FBLightConvNet in all 30. The matched-pairs rank-biserial correlation ranged from 0.29 to 1.00 with a median of 0.79, and the bootstrap 95% confidence interval excluded zero in 29 of the 30 comparisons. The single exception was FBLightConvNet versus MSVTNet on Day 3, with a mean difference of 0.95 percentage points, a 95% confidence interval of [−1.23, +3.10] and a rank-biserial correlation of 0.29. Because the correction is applied across 506 tests within each condition, it is conservative, and several comparisons whose confidence intervals exclude zero do not survive it. The effect sizes and intervals therefore indicate a more consistent advantage than the count of significant wins alone conveys, and we report both. The full pairwise results are produced by the statistical analysis script in the released code.

Because counting significant pairwise differences is not a standard ranking criterion, we also examined two conventional ones. Ranking the architectures by mean accuracy, by mean rank across participants, and by the number of significant pairwise wins selects the same seven leading architectures and gives the same first and second places, but the three criteria do not agree on the ordering of places three to five. The identity of the best-performing architecture is therefore robust to the choice of criterion, whereas the fine-grained ordering within the leading group is not and should not be over-interpreted.

### 3.4. Accuracy-Cost Trade-Off and Selection of the Optimal Architecture

Following the identification of the seven models that achieved a classification accuracy of at least 70%, we further investigated their computational cost to find an architecture that is both accurate and efficient. Table 3 presents the computational cost of these top seven models, comprising the parameter count, the multiply–accumulate operations per trial, the peak inference memory, and the measured single-trial inference time. The results show that FBLightConvNet not only achieved the highest classification accuracy of 76.94% but also had the smallest parameter count among the top-performing models. Repeating the evaluation with three random seeds did not change the ordering of the seven leading architectures, and the standard deviation across seeds did not exceed 0.55 percentage points for any of them (Table 3).

Measured cost does not follow the multiply–accumulate count. EEGSimpleConv has roughly 32 times more multiply–accumulate operations than FBLightConvNet, yet its single-trial inference time on the graphics processing unit is shorter. In absolute terms, however, these differences are small. The entire spread among the leading architectures, once EEGInceptionMI is excluded, is 2.54 ms, which is 0.025% of the 10 s trial window. Even the slowest architecture requires 44.0 ms, so every architecture meets the real-time requirement of this paradigm with a wide margin. FBLightConvNet therefore combines the highest accuracy with a computational cost comparable to that of the other leading architectures and the smallest parameter count among them, which is why we selected it as the optimal architecture for mental task classification using our ear-EEG dataset among the 23 evaluated models.

**Table 3:** Classification performance and computational cost of the seven leading deep learning models. Avg ACC is the accuracy obtained with the first seed, which is the value used elsewhere in the manuscript, and ACC over 3 seeds is the mean and standard deviation across three runs with different seed configurations. Parameter counts are trainable parameters. Multiply–accumulate operations, peak memory and inference time were measured for a single 10 s trial on the graphics processing unit described in Section 2.3, with a batch size of one and averaged over 200 timed forward passes after 30 warm-up passes.

Rank	Model	Avg ACC (%)	ACC Over 3 Seeds (%)	Num of Parameters	MACs Per Trial	Peak Memory (MB)	GPU Inference (ms)
1	FBLightConvNet [28]	76.94	76.43 ± 0.48	4834	9.20 M	11.50	4.00
2	EEGSimpleConv [27]	73.60	73.80 ± 0.31	715,706	291.20 M	14.00	1.46
3	EEGInceptionMI [29]	72.93	73.30 ± 0.49	7,144,034	14.27 G	52.10	44.00
4	IFNet [30]	72.56	72.72 ± 0.16	8578	14.10 M	12.80	2.31
5	MSVTNet [31]	72.45	72.37 ± 0.55	75,772	35.10 M	10.60	2.84
6	FBCNet [32]	70.95	71.19 ± 0.26	5474	4.60 M	14.20	2.52
7	FBMSNet [33]	70.59	70.06 ± 0.46	9893	74.20 M	15.80	3.68

### 3.5. Consistency of the Architecture Ranking Under the Original Session Split

Table 4 reports the accuracies of the seven leading architectures under the four session-wise splits. Relative to the pooled 10-fold cross-validation, the absolute accuracy falls by roughly 4 to 8 percentage points. This reduction is expected, because each model was trained on fewer trials, the test sessions were separated from the training sessions in time and in operating condition, and the session-wise splits cover Days 2 and 3 only. The ordering of the architectures is nevertheless almost unchanged. Across the 23 models the two protocols agree at Spearman ρ = 0.975 and Pearson r = 0.978 (Supplementary Figure S1). The four leading architectures occupy the same four positions, whereas the models ranked fifth to seventh change order among themselves. Within the leading group, however, the margins are smaller than the pooled cross-validation suggests. FBLightConvNet retains the highest mean accuracy at 69.16%. However, its margin over EEGSimpleConv narrows from 3.34 to 0.28 percentage points, and the two exchange places between individual splits. We therefore do not claim a reliable separation within the leading group under this protocol.

For reference, the online CSP-LDA accuracies reported in Ref. [21] for the corresponding conditions were 69.10%, 61.90%, 65.70%, and 69.50%. This comparison is informative but conservative rather than exact. The test trials are the same recordings in both cases, and the classifier was in both cases fitted only to the preceding training sessions, so the protocols correspond. The one asymmetry is that the participants received real-time feedback derived from the CSP-LDA classifier during those test sessions. Any adaptation by the participant during the session therefore favors CSP-LDA.

### 3.6. Comparison with a Conventional Machine Learning Baseline

To further validate the effectiveness of the selected architecture, FBLightConvNet, which achieved the highest accuracy among the 23 DL models, was compared against the filter-bank CSP-LDA. This algorithm is a traditional machine learning method widely recognized for its strong performance in EEG classification, and it was employed in the original study [21]. As shown in Table 5, FBLightConvNet achieved higher classification accuracies than CSP-LDA on all three recording days, and the difference was statistically significant on Days 2 and 3. Please note that the classification accuracy on Day 1 was calculated based solely on the optimal mental task pair selected for each individual subject. Under this condition, FBLightConvNet achieved a higher average accuracy of 80.74% compared to CSP-LDA’s 77.70%, although this difference did not reach statistical significance (*p* = 0.071). Nevertheless, this superior performance trend continued and became statistically significant on the subsequent days, with FBLightConvNet achieving 78.21% compared to CSP-LDA’s 70.40% on Day 2 (*p* ≤ 0.01), and 76.69% against 70.59% on Day 3 (*p* ≤ 0.001).

Because the subject-specific task pair was selected from the Day 1 data itself, the Day 1 accuracies should not be read as unbiased estimates of performance. The Day 1 CSP-LDA accuracy is the highest of the three candidate pairs evaluated on those data. On Days 2 and 3 the task pair was fixed before data collection, so these estimates are not subject to this bias. Because the selection was made with CSP-LDA, it favors the baseline rather than the deep learning models, and the advantage of FBLightConvNet on Days 2 and 3 is therefore obtained under a conservative comparison.

### 3.7. Cross-Dataset Consistency of the Architecture Ranking

**Figure 5:**
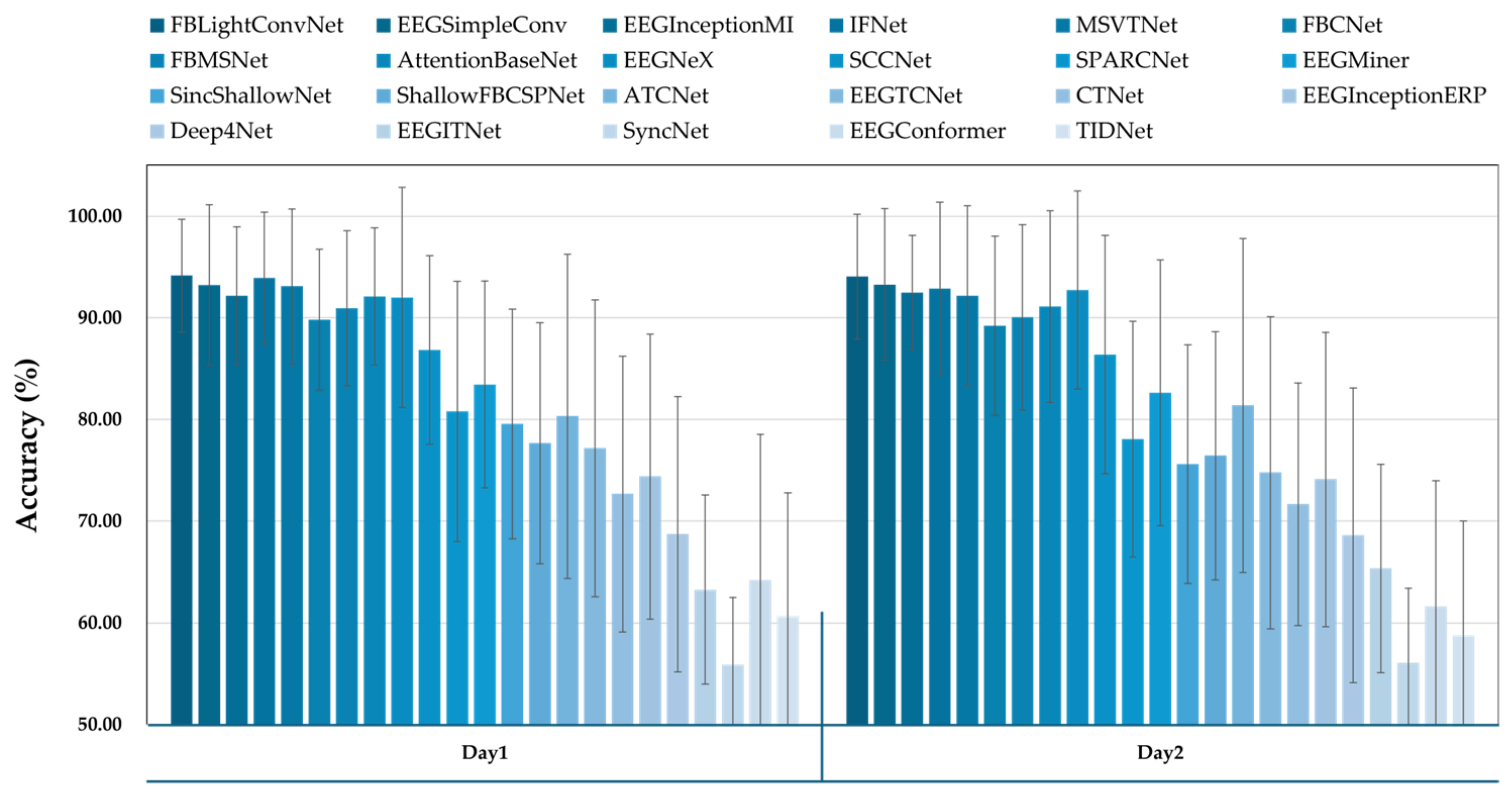
Mean classification accuracies of the 23 deep learning models on the second ear-EEG dataset [20] for each of its two recording days. Bars indicate the mean across the 30 participants and error bars indicate the standard deviation. Models are ordered by their accuracy on the main dataset. The horizontal black dotted line indicates the theoretical chance level (50%).

**Figure 6:**
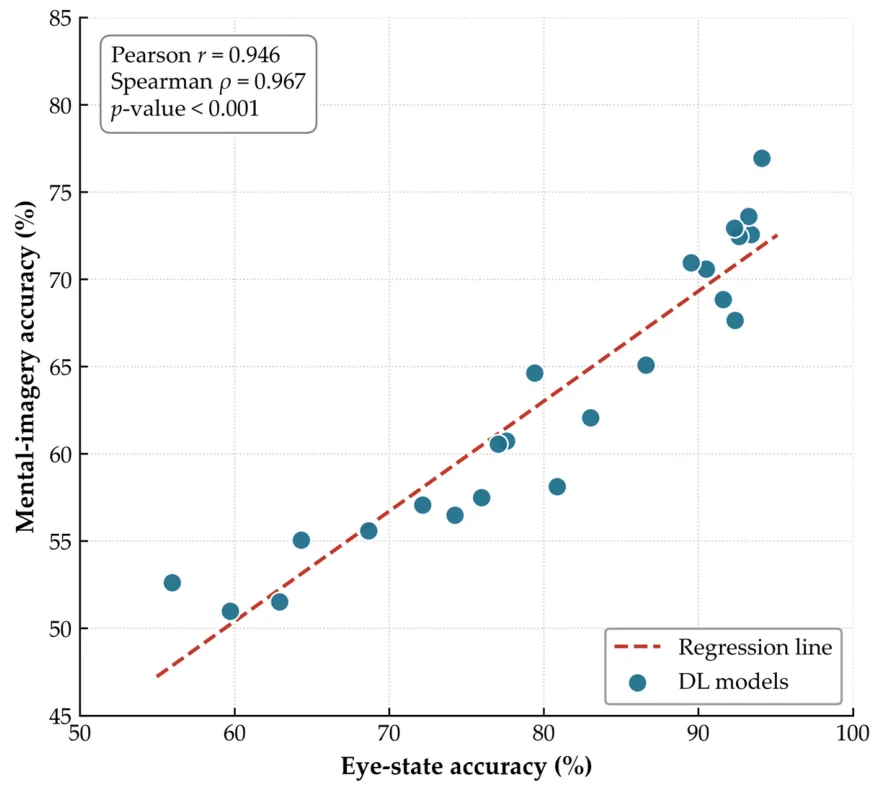
Relationship between the subject-averaged classification accuracies of the 23 deep learning models obtained on the second ear-EEG dataset [20] (*x*-axis) and on the main mental-task dataset of this study (*y*-axis). Each point denotes one model, and the dashed line is the ordinary-least-squares regression line. The Pearson and Spearman correlation coefficients computed across the 23 models are reported in the inset.

All 23 models were retrained and evaluated on the second eyes-open/eyes-closed dataset [20]. The per-architecture accuracies for each of its two recording days are shown in Figure 5. Comparing these with the subject-averaged accuracies on the main mental-task dataset (Figure 6), the accuracies on the two datasets were strongly and positively correlated (Pearson r = 0.946 and Spearman ρ = 0.967, both *p* < 0.001). FBLightConvNet achieved the highest accuracy on both datasets, and the two datasets share five of their seven leading architectures. On the second dataset, FBCNet and FBMSNet are replaced by AttentionBaseNet and EEGNeX. This indicates that the relative effectiveness of the evaluated architectures is largely preserved across different ear-EEG datasets and task paradigms. The correlation reflects the wide spread of accuracies across the full set of 23 architectures and does not establish separability among the closely ranked leading models. Because all models were retrained from scratch on the second dataset, this analysis also does not demonstrate transfer to unseen participants or validate mental-task decoding.

## 4. Discussion

This study aimed to identify which deep learning architectures developed for scalp-EEG transfer most effectively to ear-EEG mental-task classification. To this end, we analyzed the dataset of our previous study [21], in which the feasibility of decoding mental tasks from ear-EEG was established. To achieve this objective, we systematically evaluated 23 state-of-the-art DL models, originally developed for scalp-EEG, and compared them against a traditional CSP-LDA algorithm using data recorded across three distinct days. Our findings revealed that while the 23 models exhibited considerable performance variations when the data domain shifted from scalp-EEG to ear-EEG, FBLightConvNet emerged as the best architecture, achieving the highest classification accuracy with the smallest parameter count among the leading architectures. Furthermore, FBLightConvNet outperformed the conventional CSP-LDA method on all three days when evaluated with subject-specific optimal task pairs, with the difference reaching statistical significance on Days 2 and 3 (*p* ≤ 0.01 and *p* ≤ 0.001) and showing the same direction on Day 1 without reaching significance (*p* = 0.071). As noted in Section 3.6, the Day 1 task pair was selected using CSP-LDA on that same data.

Previous research on mental task classification within the ear-EEG domain have predominantly relied on traditional machine learning techniques. Among these approaches, algorithms based on CSP-LDA have been widely recognized for demonstrating meaningful performance. For example, Choi et al. demonstrated that ear-EEG data recorded during a mental arithmetic task and a light cognitive task (imagining English vocalization) could be classified with an accuracy of 78.36% using CSP-LDA [49]. However, the overall classification accuracy of ear-EEG has remained noticeably lower than that of scalp-EEG, highlighting a persistent need for performance enhancements [49]. To address this limitation, it is necessary to develop DL models, which currently show strong capabilities across various fields. Recently, a study by Han et al. demonstrated the potential of applying DL models to ear-EEG by showing that ShallowFBCSPNet, one of the 23 DL models evaluated in our study, achieved superior performance in classifying eyes-open and eyes-closed resting states compared to the conventional CSP-LDA method [20]. Despite these efforts, due to the significantly smaller volume of available data compared to the scalp-EEG domain, DL architectures specifically specified for ear-EEG have rarely been developed. Given this background, the present study represents, to the best of our knowledge, one of the first extensive attempts to apply and systematically evaluate state-of-the-art DL models, originally designed for scalp-EEG, directly within the ear-EEG domain.

The systematic performance comparison of the various DL models conducted in this study provides several meaningful insights for developing ear-EEG DL models. Table 6 presents the 23 candidate DL models in order of classification accuracy evaluated in this study, along with their average accuracy, target task, deep learning type, and input type. It should be noted that the 23 candidate models were selected to span a wide range of architectural families available within a common open-source toolbox, rather than to match the original target task of each model. Models developed for non-motor-imagery purposes (e.g., seizure detection, alcoholism recognition, emotion recognition, and ERP or SSVEP) were intentionally included so that we could examine whether transferability to ear-EEG is driven by the original target task or by architectural design. The finding that models developed for motor or mental imagery transferred best indicates that task correspondence is the primary factor, while architectural characteristics further differentiate performance among the well-matched models. First, most of the top-performing models adopted CNN-based structures. Interestingly, eight of the top ten models, including FBLightConvNet (76.94%), EEGSimpleConv (73.60%), EEGInceptionMI (72.93%), IFNet (72.56%), and FBCNet (70.95%), utilized pure CNN architectures. Conversely, models with complex hybrid structures incorporating transformer architectures tended to exhibit relatively lower performance. This pattern indicates that, in the ear-EEG setting with a limited number of channels, greater architectural complexity did not translate into higher classification accuracy. Consistent with this, FBLightConvNet achieved the highest accuracy (76.94%) with the smallest number of parameters (4834) among the top-performing models (Table 3 and Table 6), whereas several substantially larger hybrid models that incorporate transformer components ranked lower. We therefore describe this relationship in terms of the observed accuracy–cost trade-off. Based on these observations, some notable trends between classification performance and model structure can be identified. Secondly, DL models using filter bank-based inputs tended to concentrate at the top of the performance rankings. Specifically, FBLightConvNet, IFNet, FBCNet, and FBMSNet, which utilize filter bank inputs, are all placed in the top ten. This indicates that filter bank representations separated by frequency band are advantageous for effectively capturing neural oscillation patterns related to mental tasks. These findings align with existing studies demonstrating that various machine learning and deep learning models based on filter bank inputs achieve excellent performance in the scalp-EEG domain. Furthermore, they suggest that incorporating filter bank inputs can similarly lead to superior performance in deep learning models developed for ear-EEG. Lastly, DL models designed specifically for motor imagery tended to show excellent performance in classifying ear-EEG mental tasks. Notably, all seven of the top-performing models that achieved a classification accuracy above 70% were originally developed for motor imagery classification within the scalp-EEG domain. In contrast, DL models designed for other paradigms, such as emotion recognition, ERP, or SSVEP, failed to exhibit strong performance, even though all architectures received the same input. This indicates that referencing the architectural types of DL models already proven effective for similar tasks in the scalp-EEG domain can significantly benefit the development of models for ear-EEG mental task classification.

Several characteristics of the ear-EEG signal help explain the performance differences across DL architectures. Ear-EEG electrodes are located farther from the cortical sources than scalp electrodes. This reduces the signal-to-noise ratio (SNR) of task-related activity for all task types, including ERP, SSVEP, and motor or mental imagery. As a result, scalp-EEG models generally show lower accuracy on ear-EEG regardless of the target task. This tendency is consistent with previous ear-EEG studies [18,19,49]. This overall reduction, however, does not explain the differences among models. Those differences are better explained by task correspondence. Our dataset is based on mental-imagery tasks. Models originally developed for motor or mental imagery were therefore the closest match, and they performed best. In contrast, ERP- and SSVEP-oriented models target a different task type and often assume a different input format. This is consistent with our results, where EEGInceptionERP, developed for ERP and SSVEP, yielded one of the lowest accuracies (56.49%). Overall, a model’s effectiveness on ear-EEG depends not on the modality alone, but on how well its target task matches the task being decoded.

The influence of artifacts is another factor to consider when interpreting these results. In this study, the signals were processed with a 1–50 Hz band-pass filter and contralateral mean re-referencing, without a dedicated artifact-rejection procedure such as independent component analysis (ICA) or amplitude-based trial rejection. However, the same dataset was analyzed in our previous work [21], which reported clear task-related event-related desynchronization and synchronization patterns for the mental tasks. The presence of these well-defined neural responses, without the broadband high-frequency power that typically reflects muscle activity, indicates that the recordings were not dominated by artifacts. Excessive artifact contamination is therefore unlikely to be a major driver of the performance differences across models. A more detailed analysis of how artifacts affect decoding nonetheless remains a direction for future work. A useful first step would be to identify the noise types that are specific to ear-EEG, such as artifacts arising from the auricular and temporal muscles or from movement near the ear. Once these ear-EEG-specific noise characteristics are better understood, it will be important to examine whether deep learning architectures should be explicitly designed to account for them, rather than relying on models developed for scalp-EEG.

Despite these meaningful findings, this study has a limitation regarding the relatively small size of the dataset used to evaluate and compare the deep learning models. We emphasize, however, that the cross-dataset consistency reported above concerns the reproducibility of the relative ranking rather than the statistical separability of individual closely ranked models within a single dataset, which remains constrained by the modest sample size. While research in the scalp-EEG domain is active with abundant data, the ear-EEG domain remains relatively underexplored, resulting in a lack of publicly available benchmark datasets. We also note that both datasets analyzed here were acquired by our own group, so benchmarking on ear-EEG data acquired at an independent institution remains an important direction for future work. The computational cost reported here was measured on a desktop graphics processing unit, and we did not evaluate a dedicated embedded or wearable platform, so the absolute latency and memory figures should not be read as deployment measurements on such hardware. For future work, constructing a comprehensive ear-EEG benchmark dataset acquired from diverse recording devices and conducting broader comparative analyses will yield even more profound insights. Furthermore, to empirically validate the architectural design insights derived in this study, our research team plans to develop a novel deep learning model specifically tailored for ear-EEG mental task classification as a follow-up investigation.

## 5. Conclusions

To the best of our knowledge, this study is one of the first comprehensive efforts to apply and evaluate a wide range of state-of-the-art deep learning models, originally developed for the scalp-EEG domain, directly within the ear-EEG environment. By systematically comparing the classification performance and architectural characteristics of these models, our research provides design insights and a reproducible benchmarking framework. We note that these findings are derived from a limited dataset and therefore require further validation. Nonetheless, we expect them to serve as a useful reference for future researchers aiming to design deep learning architectures tailored for ear-EEG-based mental task classification.

## Figures and Tables

**Table 1:** Optimal mental task pairs selected for each subject on Day 1.

Optimal Mental Task Pair	Subject ID														
	1	2	3	4	5	6	7	8	9	10	11	12	13	14	Total
MA vs. MS	✓		✓			✓		✓	✓		✓		✓		7
MA vs. WA		✓								✓		✓		✓	4
MS vs. WA				✓	✓		✓								3

**Table 2:** Representative example of pairwise statistical comparisons of the 23 DL models for the MA vs. WA task condition on Day 1. For the ‘Total’ column, any significant outperformance (*p* ≤ 0.05) is counted as 1, regardless of the specific significance level. The numbers from 1 to 23 in the columns correspond to the respective DL models listed in the rows. Asterisks denote the significance level of each pairwise difference (* *p* ≤ 0.05, ** *p* ≤ 0.01).

MA vs. WA (Day 1)	1	2	3	4	5	6	7	8	9	10	11	12	13	14	15	16	17	18	19	20	21	22	23	Total
FBLightConvNet [28]		*	**	**	*	**	**	**	**	**	**	**	**	**	**	**	**	**	**	**	**	**	**	22
EEGSimpleConv [27]									*	*	*	*	*	*	**	**	**	**	*	**	**	**	**	15
EEGInceptionMI [29]									*	*	**	**	**	*	**	**	**	**	**	**	**	**	**	15
IFNet [30]									*	*	**	*	**	*	**	**	**	**	**	**	**	**	**	15
MSVTNet [31]										*	*	*	*	*	*	**	*	**	**	**	**	**	**	14
FBCNet [32]											*	*	*	*	*	*	*	**	*	*	**	**	**	13
FBMSNet [33]											*	*	*	*	*	*	*	**	*	**	**	**	**	13
AttentionBaseNet [34]											*	*	**	*	**	**	*	**	**	**	**	**	**	13
SCCNet [36]												*	*		*	*	*	**	**	**	**	**	**	11
EEGNeX [35]															*	*	*	**		*	**	*	*	8
SPARCNet [39]															*	*	*	**		**	**	*	**	8
SincShallowNet [38]																		*		*	**	*	*	5
ShallowFBCSPNet [26]																		*		*	**	*	**	5
EEGMiner [37]																				*			*	2
Deep4Net [26]																						*		1
EEGTCNet [41]																								0
CTNet [42]																								0
EEGInceptionERP [43]																								0
ATCNet [40]																								0
EEGITNet [44]																								0
SyncNet [47]																								0
EEGConformer [45]																								0
TIDNet [46]																								0

**Table 4:** Classification accuracies of the seven leading architectures under the original training and test session splits of Ref. [21], together with the pooled cross-validation accuracies from Table 3. Split 1 trained on Day 2 sessions 1–3 and tested on Day 2 sessions 4–5. Split 2 trained on Day 2 sessions 1–3 and tested on Day 3 sessions 4–5. Split 3 trained on Day 3 sessions 1–3 and tested on Day 3 sessions 6–7. Split 4 trained on the Day 2 and Day 3 training sessions and tested on Day 3 sessions 8–9. The pooled cross-validation accuracies were computed over the three binary task pairs on Day 1 and the subject-specific optimal pair on Days 2 and 3, whereas the session-wise splits cover Days 2 and 3 only and use the subject-specific optimal task pair. The online CSP-LDA accuracies reported in Ref. [21] are given for reference. The Difference column gives the reduction in accuracy from the pooled cross-validation to the mean of the four session-wise splits.

Model	Split 1	Split 2	Split 3	Split 4	Mean	Pooled 10-Fold CV	Difference
FBLightConvNet [28]	73.93	66.43	67.86	68.42	69.16	76.94	7.78
EEGSimpleConv [27]	71.61	63.57	70.00	70.35	68.88	73.60	4.72
EEGInceptionMI [29]	68.75	65.71	69.11	70.16	68.43	72.93	4.50
IFNet [30]	71.07	64.46	66.07	68.39	67.50	72.56	5.06
MSVTNet [31]	65.89	62.50	66.96	68.21	65.89	72.45	6.56
FBCNet [32]	66.79	65.18	65.00	69.12	66.52	70.95	4.43
FBMSNet [33]	69.29	63.04	64.46	68.04	66.21	70.59	4.38
CSP-LDA [21]	69.10	61.90	65.70	69.50	66.55	70.63	4.08

**Table 5:** Comparison of mean classification accuracies between CSP-LDA and FBLightConvNet using the optimal mental task pair for each subject across three recording days.

Subject No.	Task Pair	Day 1		Day 2		Day 3	
		CSP-LDA	FBLightConv.	CSP-LDA	FBLightConv.	CSP-LDA	FBLightConv.
1	MA vs. MS	55.20	72.00	48.80	70.00	53.80	57.78
2	MA vs. WA	80.60	84.00	66.10	63.00	71.90	80.56
3	MA vs. MS	80.80	71.81	74.60	74.00	76.40	78.89
4	MS vs. WA	75.60	84.00	72.80	82.00	86.20	93.33
5	MS vs. WA	77.60	69.00	67.60	73.00	53.80	59.44
6	MA vs. MS	97.70	99.00	87.80	93.00	89.70	88.89
7	MS vs. WA	65.40	64.00	52.10	67.00	49.90	56.11
8	MA vs. MS	87.10	92.50	80.90	83.00	73.90	93.89
9	MA vs. MS	71.10	87.00	76.80	85.00	73.40	71.67
10	MA vs. WA	81.50	91.00	95.40	97.00	96.30	98.33
11	MA vs. MS	91.50	86.00	82.10	97.00	79.20	83.69
12	MA vs. WA	82.90	84.00	74.60	84.00	76.50	90.56
13	MA vs. MS	69.60	64.00	57.20	66.00	53.20	57.22
14	MA vs. WA	71.20	82.00	48.80	61.00	54.10	63.33
Average		77.70	80.74	70.40	78.21 **	70.59	76.69 ***
STD		10.93	10.86	14.48	12.27	15.19	15.47

**: *p* ≤ 0.01; ***: *p* ≤ 0.001.

**Table 6:** Summary of the 23 candidate deep learning models evaluated in this study, including their average classification accuracies, target tasks, deep learning types, and input types.

Rank	Model	Avg ACC (%)	Target Task	Deep Learning Type	Input Type
1	FBLightConvNet [28]	76.94	Motor Imagery	CNN	Filter Bank
2	EEGSimpleConv [27]	73.60	Motor Imagery	CNN	Raw Data
3	EEGInceptionMI [29]	72.93	Motor Imagery	CNN	Raw Data
4	IFNet [30]	72.56	Motor Imagery	CNN	Filter Bank
5	MSVTNet [31]	72.45	Motor Imagery	CNN + RNN + Transformer	Raw Data
6	FBCNet [32]	70.95	Motor Imagery	CNN	Filter Bank
7	FBMSNet [33]	70.59	Motor Imagery	CNN	Filter Bank
8	AttentionBaseNet [34]	68.83	Motor Imagery	CNN + Transformer	Raw Data
9	EEGNeX [35]	67.65	Motor Imagery	CNN	Raw Data
10	SCCNet [36]	65.08	Motor Imagery	CNN	Raw Data
11	SPARCNet [39]	64.62	Epilepsy	CNN	Raw Data
12	EEGMiner [37]	62.07	Emotion Recognition	CNN	Raw Data
13	SincShallowNet [38]	60.73	Motor Imagery	CNN	Raw Data
14	ShallowFBCSPNet [26]	60.56	General	CNN	Raw Data
15	ATCNet [40]	58.11	General	CNN + RNN + Transformer	Raw Data
16	EEGTCNet [41]	57.49	Motor Imagery	CNN + RNN	Raw Data
17	CTNet [42]	57.06	Motor Imagery	CNN + Transformer	Raw Data
18	EEGInceptionERP [43]	56.49	ERP, SSVEP	CNN	Raw Data
19	Deep4Net [26]	55.58	General	CNN	Raw Data
20	EEGITNet [44]	55.05	Motor Imagery	CNN + RNN	Raw Data
21	SyncNet [47]	52.61	Recognition Alcoholism	CNN	Raw Data
22	EEGConformer [45]	51.51	General	CNN + Transformer	Raw Data
23	TIDNet [46]	50.98	General	CNN	Raw Data

## Data Availability

The datasets used in this study are available from the corresponding author upon reasonable request, provided they are utilized for appropriate research purposes. The analysis code and the configuration files required to reproduce the comparison are publicly available at https://github.com/toiro2000/Ear-EEG-DL-Benchmark (accessed on 31 July 2026) and archived at https://doi.org/10.5281/zenodo.21716287.

## Supplementary Materials

The following supporting information can be downloaded at: https://www.mdpi.com/article/10.3390/bios16080437/s1, Figure S1: Relationship between the subject-averaged classification accuracies of the 23 deep learning models obtained under the pooled 10-fold cross-validation (*y*-axis) and under the original training and test session split (*x*-axis). Each point denotes one model, and the dashed line is the ordinary-least-squares regression line. The Pearson and Spearman correlation coefficients computed across the 23 models are reported in the inset.

## Abbreviations

The following abbreviations are used in this manuscript:

BCI	Brain–computer interface
SNR	Signal-to-noise ratio
DNN	Deep neural network
CSP-LDA	Common spatial pattern-linear discriminant analysis
SSVEP	Steady-state visual evoked potential
EEG	Electroencephalography
ML	Machine learning
CNN	Convolutional neural network
MA	Mental arithmetic
MS	Mental singing
WA	Word association
