# Supplementary material for: Deep Learning for Ear-EEG-Based Brain–Computer Interface: A Systematic Comparison and Design Insights
Source: Biosensors (Basel). 2026 Aug 12;16(8):437. doi: 10.3390/bios16080437 (PMC13510306; doi:10.3390/bios16080437)

biosensors

Figure S1. Relationship between the subject-averaged classification accuracies of the 23 deep learning models obtained under the pooled 10-fold cross-validation (y-axis) and under the original training and test session split (x-axis). Each point denotes one model, and the dashed line is the ordinary-least-squares regression line. The Pearson and Spearman correlation coefficients computed across the 23 models are reported in the inset.

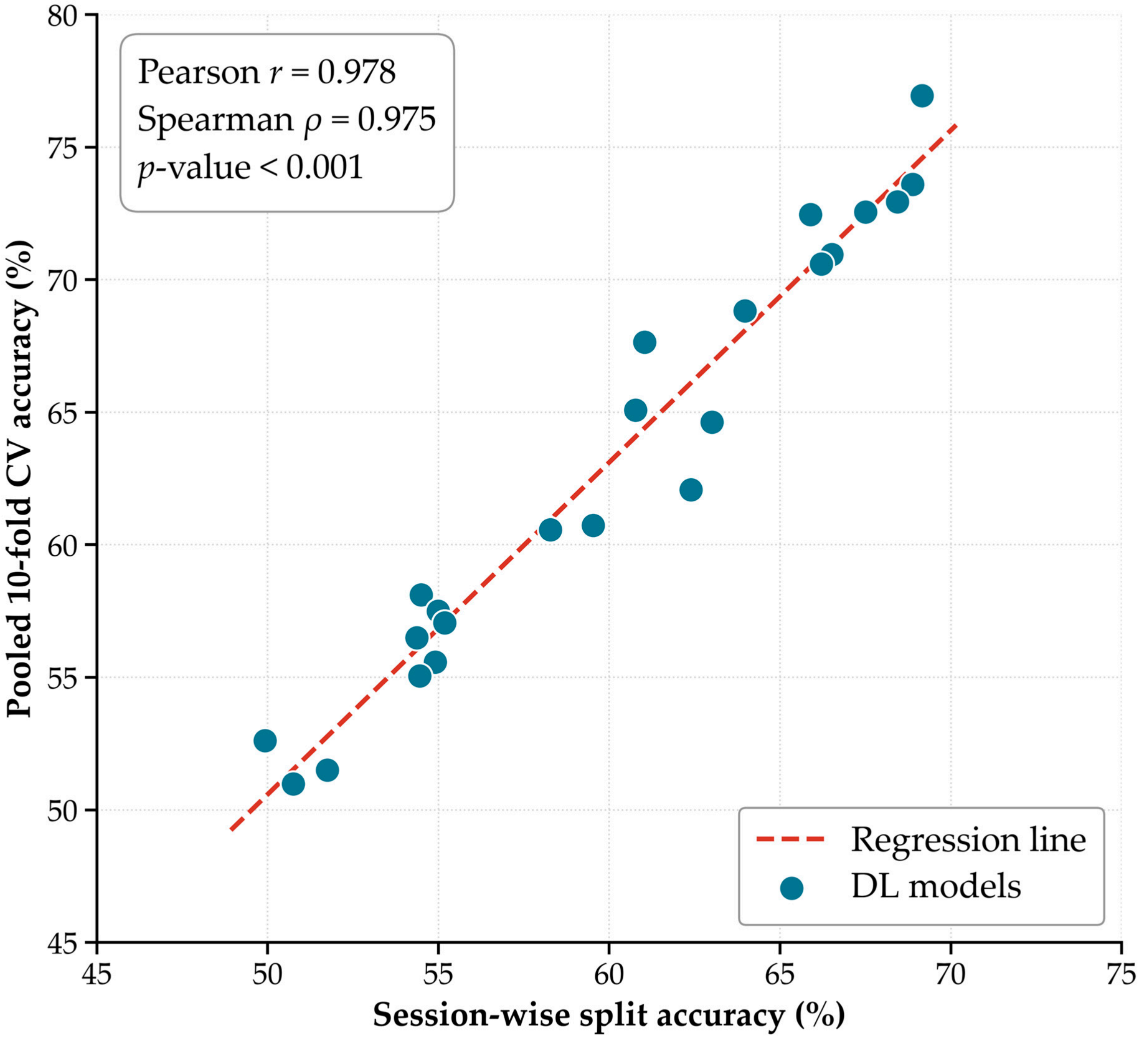

Supplement: Supplementary file 1 [file biosensors-16-00437-s001.zip › biosensors-4445328-supplementary.pdf]
